# Fracture Load in Monolithic Multilayer Zirconia Crowns From Three Manufacturers: an in Vitro Study

**DOI:** 10.34172/joddd.44548

**Published:** 2026-06-30

**Authors:** Neil Duvan Gutierrez Echavarria, Juan Jose Perico Gomez, Camilo Andres Galvez Reyes, Alejandra Marleth Ordoñez Molina, Julian Andres Tamayo Cardona

**Affiliations:** ^1^Department of Oral Rehabilitation, School of Dentistry, Institución Universitaria Colegios de Colombia, UNICOC, Bogotá, Colombia; ^2^Department of Research, Institución Universitaria Colegios de Colombia, UNICOC, Bogotá, Colombia

**Keywords:** Dental crown, Fracture load, Mechanical properties, Multilayer monolithic zirconia

## Abstract

**Introduction:**

Multilayer zirconia crowns have gained popularity in oral rehabilitation due to their combination of gradient aesthetics and mechanical strength. However, gaps exist in the literature regarding differences in fracture load among commercial brands, a critical variable for their selection in patients with parafunctional habits. Previous studies have evaluated monolithic zirconia, but few have examined multilayer zirconia under standardized conditions. The present in vitro study compared the fracture load of monolithic crowns fabricated from multilayer zirconia discs of three commercial brands: BeautyZir, Upcera, and Yucera.

**Methods:**

A comparative experimental study was conducted using 30 monolithic crowns made from multilayer zirconia discs, distributed as follows: 10 crowns from ST + Multilayer® BeautyZir, 10 from 4D Pro® Yucera, and 10 from Explore® Upcera. Abutments were fabricated with a chamfer finish preparation on an ivorine tooth (#16) and scanned with the TRIOS 3 intraoral system (3Shape®). The resulting STL file was laser-sintered in cobalt‒chromium to obtain the abutment for crown placement. Crown design was performed in Exocad® software with a central fossa depth of 2.1 mm. The crowns were milled according to each brand’s specifications and subjected to fracture load testing on a universal Instron® machine, applying axial force using a 12-mm-diameter metallic sphere at a crosshead speed of 3 mm/s. Data were collected using Excel and analyzed using one-way ANOVA and post hoc Tukey tests (α=0.05), following verification of normality with the Kolmogorov-Smirnov and Shapiro-Wilk tests.

**Results:**

Mean fracture loads (±SD) were: ST + Multilayer®: 1807 N (±463), Explore®: 1682 N (±368), Yucera®: 1173 N (±183). ANOVA revealed significant differences between groups (*P*=0.001). The Tukey test showed significant differences between Yucera and Upcera (*P*=0.010) and Yucera and BeautyZir (*P*=0.001), with Upcera and BeautyZir demonstrating significantly higher fracture loads than Yucera; however, no differences were observed between Upcera and BeautyZir (*P*=0.719). All the tested brands exceeded physiological masticatory forces (133–727 N), but Yucera had the lowest safety margin. The high standard deviation in BeautyZir (±463 N) suggests variability in manufacturing quality.

**Conclusion:**

The three multilayer zirconia brands tested withstood normal occlusal forces, but Upcera and BeautyZir were better suited for patients at risk of higher loads. The lack of difference between Upcera and BeautyZir supports their interchangeable clinical use. The lower resistance of Yucera and high dispersion in BeautyZir emphasize the need for stringent quality control during manufacturing. Future studies should incorporate thermocycling, cementation, and in vivo follow-up to validate these findings under clinical conditions.

## Graphical Abstract

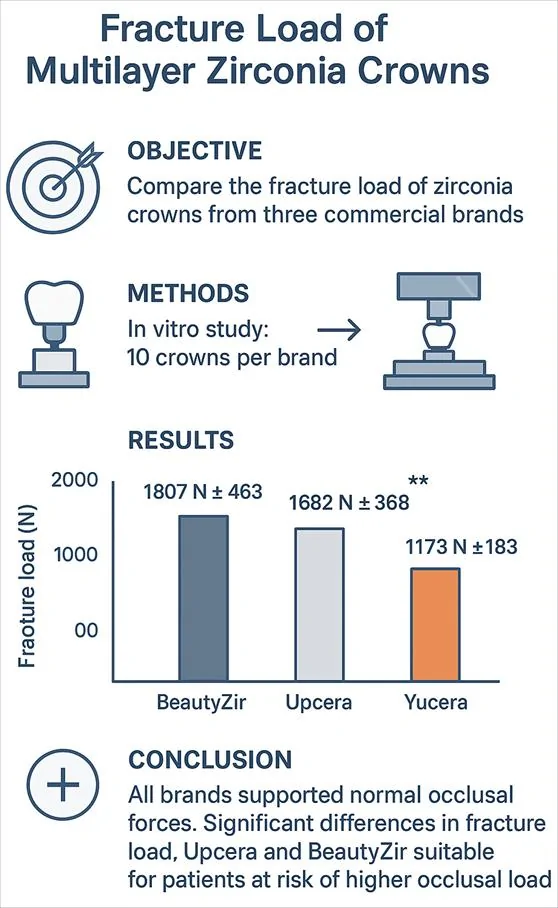

## Introduction

 The evolution of dental materials has enabled increasingly aesthetic and functional restorations. In oral rehabilitation, monolithic zirconia crowns have become a reliable alternative due to their high mechanical strength, biocompatibility, and good optical properties.^1,2^ Among these material variations, multilayer zirconia stands out for offering improved color transition without compromising restoration structural integrity.^3,4^

 However, multiple commercial brands offer multilayer zirconia discs with proprietary characteristics and manufacturing technologies, which may affect the final mechanical behavior of restorations.^4^ A critical factor to evaluate in these materials is fracture load, defined as the maximum force a crown can withstand before fracturing, essential to ensure the clinical longevity of restorations, especially in posterior high-load regions.

 The mechanical properties of restorative materials are fundamental to their ability to resist masticatory forces on teeth. These forces range between 133 N and 727 N,with molars bearing the highest occlusal load (approximately 600‒750 N), followed by canines and incisors, with forces of 350 N and 200 N, respectively.^5^ Therefore, posterior restorations must resist significant compressive forces, necessitating adequate restorative material selection to guarantee clinical durability under functional stress.

 In recent years, thanks to advances in adhesive systems, ceramics—particularly zirconia—have gained popularity for their excellent mechanical properties and high strength compared to glass matrix ceramics.^6,7^ Initially, zirconia had aesthetic limitations compared to other ceramics,^7^ prompting clinicians to use additional techniques such as layering with veneering ceramics. However, these increased the risk of failures such as chipping.^8,9^ The introduction of multilayer zirconia discs sought to enhance aesthetics directly through the material via internal color and translucency gradation.^3,9,10^

 Oral rehabilitation with prostheses fabricated from zirconia has established itself as an optimal alternative due to its durability and aesthetics, especially in populations with high edentulism prevalence. In Colombia, this public health problem is critical: according to ENSAB IV,^11^ 41.19% of the population has partial edentulism, with an incidence of 12.17 cases per 100,000 inhabitants. This situation worsens with increasing life expectancy, as aging correlates with cumulative tooth loss.^12^ This clinical reality demands restorative solutions combining mechanical strength to withstand decades of function and aesthetics to preserve orofacial harmony, intrinsic characteristics of multilayer zirconia.

 However, multilayer zirconia compositions and manufacturing technologies vary by manufacturer, potentially influencing clinical performance,^4^ particularly fracture load, understood as the maximum force a restoration can endure before fracturing. The lack of independent, comparative studies on this parameter across commercial multilayer zirconia brands significantly limits clinical decision-making, potentially leading to inadequate material selection and rehabilitation failure.^13^

 Based on the above, it is necessary to generate objective, conflict-free evidence on the mechanical performance of multilayer zirconia discs available on the market to optimize clinical oral rehabilitation practice and improve long-term functional and aesthetic outcomes.

## Methods

###  Study Design

 This study employed a comparative in vitro experimental design to evaluate the fracture load of monolithic crowns fabricated from multilayer zirconia by three commercial brands.

###  Specimens 

 Thirty monolithic crowns made from multilayer zirconia discs were distributed as follows: 10 crowns manufactured from ST + Multilayer® BeautyZir, 10 crowns from 4D Plus® Yucera, and 10 crowns from Explore® Upcera.

###  Sampling

 A statistical power analysis was conducted using G*Power software (version 3.1.9.7), with the following parameters:

- Significance level (α): 0.05 (5% Type I error probability)
- Statistical power (1-β): 95% (probability of detecting differences if they exist)
- Effect size (f): 0.30 (based on similar previous multilayer zirconia studies)
- Number of groups: 3.

The calculation yielded a minimum sample size of 9 samples per group. To increase robustness, mitigate possible losses due to manufacturing defects, and align with previous studies, a sample size of n = 10 per group was established.

###  Abutment and Crown Fabrication 

 An ivorine tooth (#16) was prepared with a 360° full crown preparation using a chamfer finish line guided by a condensation silicone matrix (Protesil Labor®, Vanini Dental Industries), pre-fabricated to preserve the original anatomy and control axial and occlusal wear. The prepared model was scanned using an intraoral TRIOS 3 Wireless® scanner (3Shape, Denmark), which generated an STL file. Using this file, a digital support abutment was designed with CAD software and fabricated by Phibo® (Colombia) using laser sintering of a cobalt‒chromium (CoCr) alloy (Figure 1).

**Figure 1:**
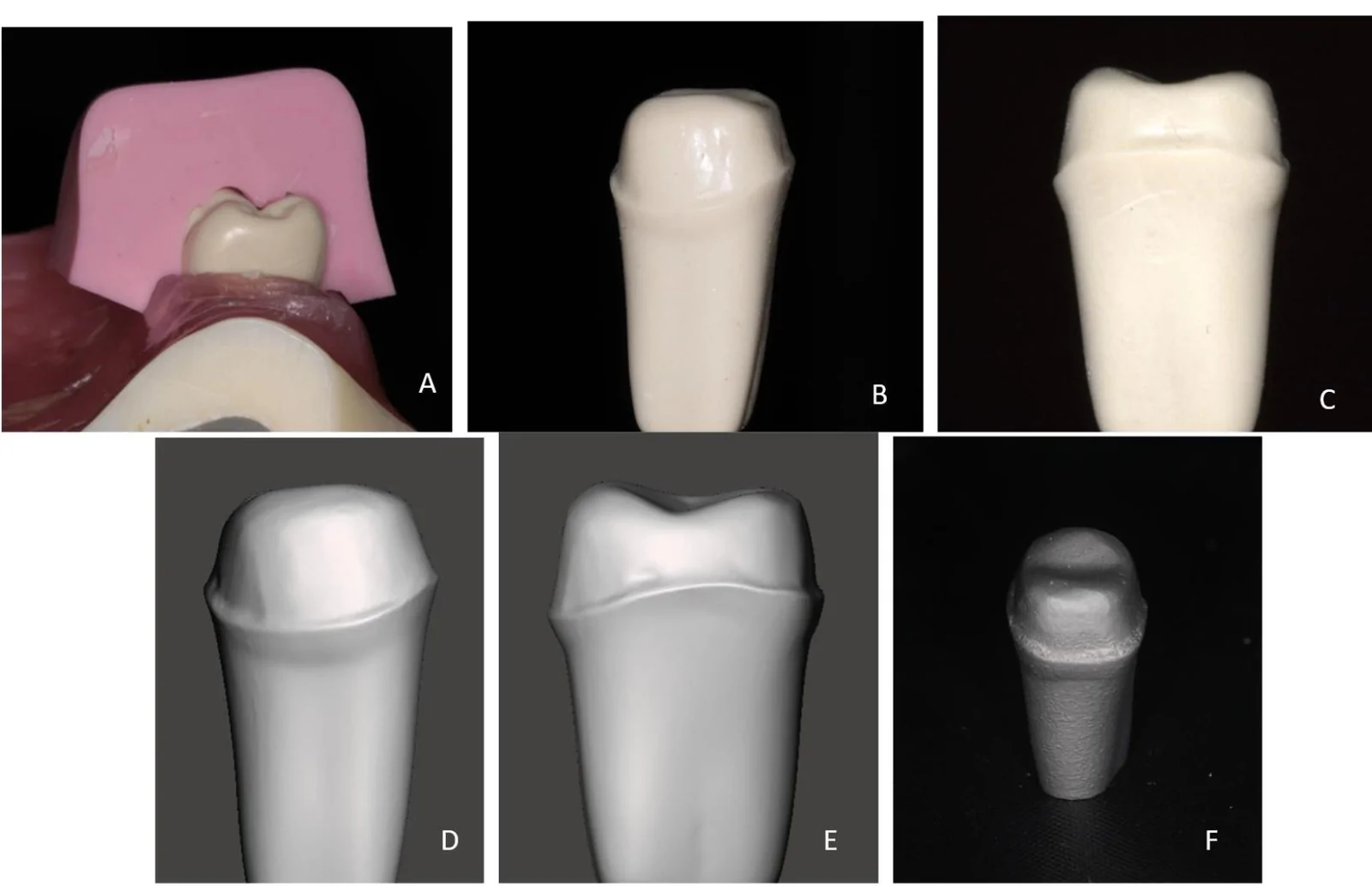
(A) Silicone matrix on ivorine model. (B) Buccal view of abutment preparation on ivorine model. (C) Mesial view of abutment preparation on ivorine model. (D) Buccal view of scanned model. (E) Mesial view of scanned model. (F) Abutment fabricated by laser sintering

 The design of the monolithic crowns was performed using the CAD software DentalCAD 3.2 Elefsina® (Exocad) (Figure 2). Subsequently, CAM machining was performed on a five-axis milling machine, Upcera A51®, using 0.6 mm burs. After milling, the frameworks underwent sintering in the Zirkonofen 600® furnace (Zirkonzahn, Italy), following the thermal protocols recommended by each manufacturer for their respective multilayer zirconia.

**Figure 2:**
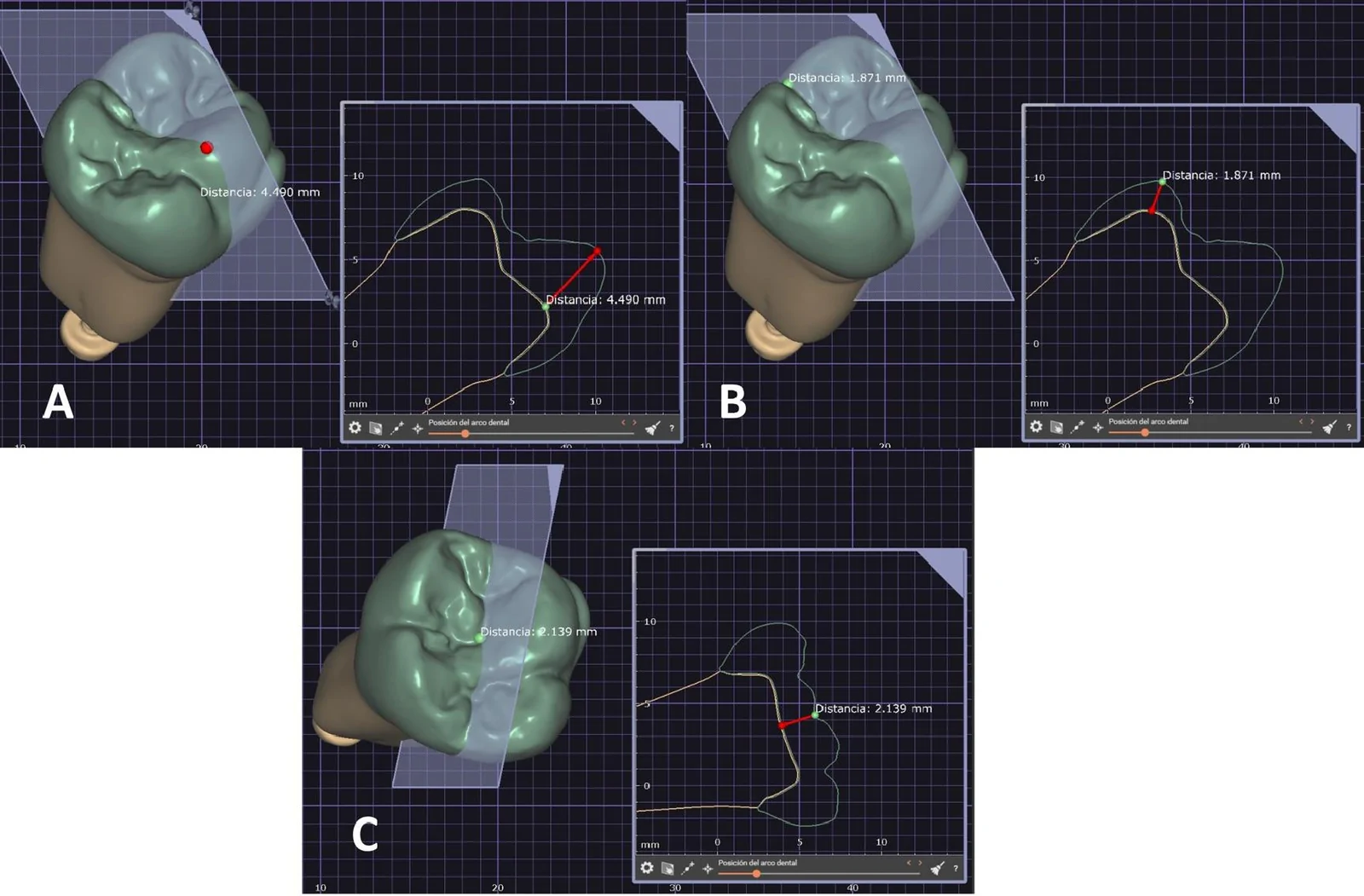
(A) Crown design in Exocad software showing measurement of mesiopalatal cusp. (B) Crown design in Exocad software showing measurement of mesiovestibular cusp. (C) Crown design in Exocad software measuring distance from abutment to central fossa

###  Fracture Load Testing 

 Each crown was manually seated on its respective metal abutment, ensuring passive fit without adhesive intervention. For the mechanical test, a 12-mm-diameter metal sphere was used to distribute the applied load evenly over the occlusal surface. The sphere was positioned on the central fossa of each crown, aligning the load axis perpendicular to the occlusal plane. Subsequently, the abutment‒crown assembly was fixed in a universal testing machine (Instron®), and a progressive axial load was applied at a constant crosshead speed of 3 mm/s until complete restoration fracture (Figure 3).

**Figure 3:**
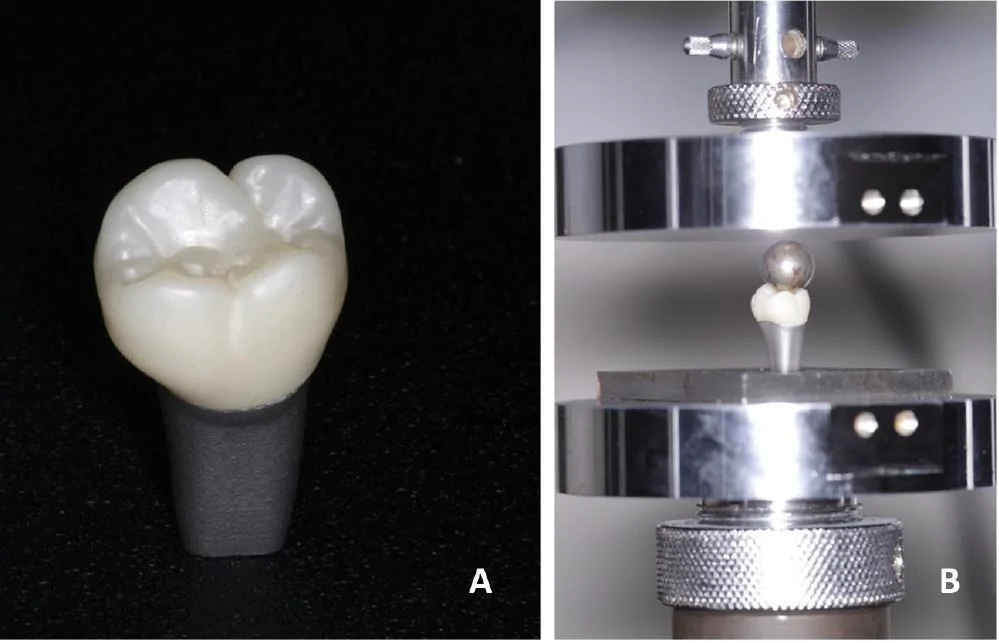
(A) Monolithic multilayer zirconia crown mounted on metallic abutment, ready for fracture load testing. (B) Metallic sphere applying load during fracture testing with an INSTRON universal testing machine

 The values corresponding to the maximum force applied (in Newtons) before the fracture of each crown were recorded in a Microsoft Excel® spreadsheet (Microsoft Corp., USA). Prior to conducting the definitive tests, a pilot protocol was performed to standardize the experimental procedure and ensure reproducibility of loading conditions for the three commercial brands evaluated.

###  Statistical Analysis

 The data were analyzed using IBM SPSS Statistics version 26 (IBM Corp., Armonk, NY, USA). Normality of the distribution was assessed using the Kolmogorov-Smirnov (*P* = 0.200) and Shapiro-Wilk (*P* = 0.075) tests, both of which yielded *P*-values > 0.05, confirming a normal distribution of the data. Based on these results, one-way ANOVA was applied to compare mean fracture loads between the three experimental groups. Upon identifying statistically significant differences via ANOVA, post hoc Tukey tests were conducted to determine which pairs of groups differed significantly.

###  Ethical Considerations

 In accordance with Resolution 8430 of 1993 from the Colombian Ministry of Health, which establishes scientific, technical, and administrative norms for health research, this study was classified as risk-free because it involved no direct intervention or interaction with human subjects and no use of sensitive or identifiable personal information.

## Results

Table 1 shows the individual fracture load values (in Newtons) obtained for the monolithic crowns fabricated with multilayer zirconia from three commercial manufacturers. The highest recorded value corresponded to sample 1 of the BeautyZir® group, while the lowest value was observed in sample 5 of the Yucera® group.

**Table 1:** Fracture load values (Newtons) for the tested crowns

	**1**	**2**	**3**	**4**	**5**	**6**	**7**	**8**	**9**	**10**
*BeautyZir*	2357	2679	2093	1955	1762	1195	1408	1607	1534	1475
*Upcera*	1568	1064	2284	1960	1744	1696	1236	2082	1553	1633
*Yucera*	1193	1140	1008	1026	993	1184	1324	1008	1569	1282

Table 2 summarizes the measures of central tendency and dispersion corresponding to the fracture load (in Newtons) for each experimental group. Although the BeautyZir® group recorded the highest average values, it also presented the highest standard deviation, indicating greater variability among its samples. In contrast, the Yucera® group showed the lowest average values but with a smaller standard deviation, suggesting greater homogeneity and possibly higher predictability in its mechanical behavior. Figure 4 shows the medians, quartiles, and outliers for each group.

**Table 2:** Summary of central tendency and dispersion measures for fracture load (Newtons)

**Brand**	**Minimum**	**Maximum**	**Median**	**Mean (SD)**	**25th Percentile**	**75th Percentile**
*Yucera*	993	1569	1162	1173 (± 183)	1008	1282
*Upcera*	1064	2284	1665	1682 (± 368)	1553	1960
*BeautiZir*	1195	2679	1685	1807 (± 463)	1475	2093

**Figure 4:**
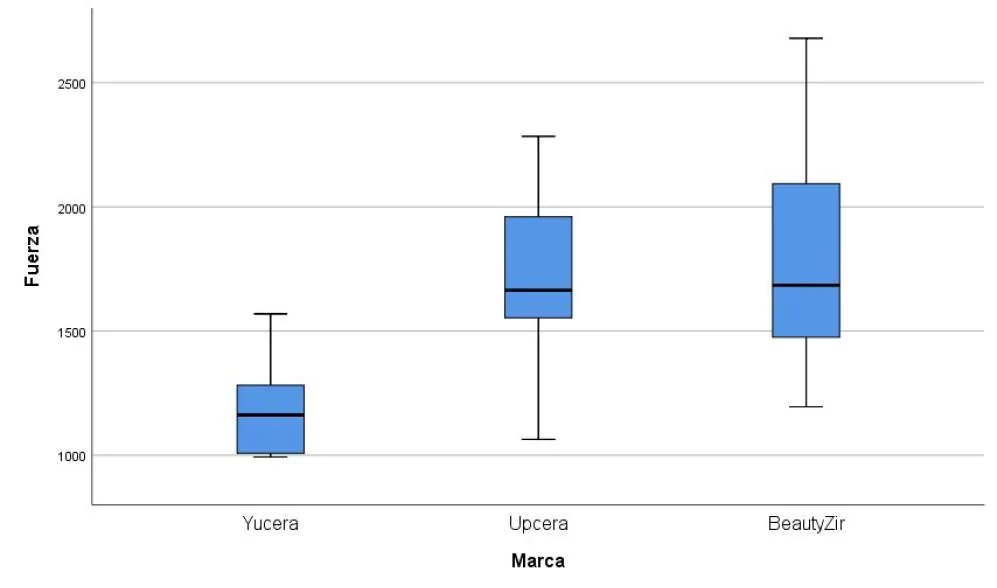
Box-and-whisker plot showing the distribution of fracture loads among crowns fabricated from three commercial multilayer zirconia brands

 The comparison of mean fracture loads between the three experimental groups was performed using one-way ANOVA, which revealed statistically significant differences between the evaluated brands (*P* = 0.001). Tukey HSD tests were used as a post hoc analysis to identify specific pairs of groups that differed significantly.

Table 3 summarizes the results of this test, revealing significant differences between Yucera® and Upcera® (*P* = 0.010) and between Yucera® and BeautyZir® (*P* = 0.001). No significant differences were observed between Upcera® and BeautyZir® (*P* = 0.719).

**Table 3:** Results of post hoc Tukey tests for multiple comparisons of fracture load between groups

**Brands**		**Mean Difference**	**Std. Error**	**Sig.**	**95% Confidence Interval**	
					**Lower Limit**	**Upper Limit**
*Yucera*	Upcera	-509.300^*^	159,752	0,010	-905,39	-113,21
	BeautyZir	-633.800^*^	159,752	0,001	-1029,89	-237,71
*Upcera*	Yucera	509.300^*^	159,752	0,010	113,21	905,39
	BeautyZir	-124,500	159,752	0,719	-520,59	271,59
*BeautyZir*	Yucera	633.800^*^	159,752	0,001	237,71	1029,89
	Upcera	124,500	159,752	0,719	-271,59	520,59

## Discussion

 The results of this study showed statistically significant differences** (***P* = 0.001) in the fracture load of monolithic crowns made with multilayer zirconia between the three evaluated commercial brands. The highest average values were recorded for BeautyZir® (1807 ± 463 N) and Upcera® (1682 ± 368 N), suggesting better mechanical performance than Yucera®, which showed the lowest average value (1173 ± 183 N). Overall, the mean fracture loads ranged between 1173 N and 1807 N. These findings are consistent with previous studies highlighting the influence of factors such as chemical composition, structural design of restorations—for example, preparation and crown design—and manufacturing protocols—for example, cooling and sintering times—on the mechanical strength of multilayer zirconia.^4,14-16^ The observed differences reinforce the need to consider not only the commercial brand but also microstructural properties and quality control when selecting this material for clinical use.

 The study conducted by Ting Sun et al.^15^ highlights that both the crown thickness and its structural design are critical factors for ensuring integrity under occlusal loading conditions. In the present work, a depth of 2.1 mm was standardized in the central fossa—the area where force was applied—within the range recommended by Sun et al.^15^ to balance strength and aesthetics. However, the statistically significant differences between groups (*P* = 0.001) suggest that, beyond thickness, the layer arrangement in multilayer zirconia disks and the intrinsic properties of each disk play decisive roles. This approach aligns with the findings reported by Xuejing Li et al.,^17^ who demonstrated that milling depth in multilayer disks affects strength: layers located closer to the “dentin” zone of the disk—that is, the innermost layers—offer greater fracture load than those near the enamel-like surface. Although the present study did not control the block orientation during milling, it is plausible that inter-brand variations in this aspect may contribute to the observed results, particularly in the Yucera® group, which showed the lowest average strength (1173 N).

 Technical data reported in the Safety Data Sheets (SDS) from the manufacturers reveal a structural difference: ST+Multilayer from BeautyZir® and Explore from Upcera® use systems with 5 gradual layers, while 4D pro from Yucera® uses an architecture of 8 thinner layers. This increased stratification in Yucera® aims to optimize aesthetic translucency by increasing yttria concentrations in the superficial layers, thereby promoting a translucent cubic phase. However, this aesthetic advantage comes at a mechanical cost: on one hand, increasing yttria content reduces tetragonal phase transformation, a key mechanism for material toughness;^16^ furthermore, each interface between layers acts as a stress concentrator due to differing thermal coefficients between layers, inducing microcracks during sintering; finally, the higher proportion of cubic phase decreases intrinsic strength. These factors synergistically explain Yucera®’s lower fracture load, showing that strategies to maximize aesthetics through elaborate multilayers compromise biomechanical integrity in areas of high occlusal stress.

 Junichi Honda et al.^18^ reported notably higher fracture loads (6610 N) in multilayer zirconia crowns (Katana Zirconia ML) on titanium implants. In their study, crowns with a uniform thickness of 2.5 mm were designed with a 2.7-mm-diameter hole for implant abutment adaptation, simulating realistic occlusal conditions. Despite this geometric discontinuity, the high recorded strength suggests that increased thickness (2.5 mm) and the multicapa material characteristics compensated for potential fracture points. These findings contrast with studies evaluating thinner crowns (1.5 mm), such as Sun et al.,^15^ where the maximum load was 4109.9 N. The differences underline not only the direct relationship between thickness and strength, but also the impact of structural design and multilayer material technologies on biomechanical performance. However, these discrepancies could also be attributed to key methodological differences, such as the use of titanium abutments, which may distribute forces better than the non-cemented cobalt‒chromium abutments used in our study. Cement acts as a tension buffer,^19^ which could explain the higher strength; additionally, their protocol included artificial aging, whereas our study assessed immediate post-manufacturing strength. Honda et al.’s^18^ crowns had a thickness of 2.5 mm compared to 2.1 mm in the present study, which, according to Sun et al.,^15^ would further increase strength.

 In studies of monolithic crowns made from non-multilayer zirconia, such as that of Takashi Nakamura et al.,^20^ extremely high fracture loads were recorded (10,610 N), possibly due to the structural homogeneity of monolithic zirconia. Nevertheless, protocols for cementing crowns to abutments, loading speed (0.5 mm/s), and the smaller force application sphere (7 mm) make direct comparison difficult. On the other hand, Keisuke Nakamura et al.^21^ reported values closer to those of the present study (1669 ± 311 N) in monolithic crowns 0.6 mm thick on resin abutments, using a 6-mm sphere. The similarity suggests that while multilayer zirconia offers aesthetic advantages, its strength may be lower than that of non-multilayer monolithic zirconia in certain clinical scenarios.

 The results of the present study align with previous research analyzing critical factors in zirconia strength. Abdulmajeed et al.^9^ observed that the yttria concentration in zirconia formulations directly influences fracture load, as variations in this component alter the stability of the tetragonal phase, which is key to stress transformation and material toughness. Furthermore, that study, which used multilayer zirconia disks similar to those evaluated here, showed thickness as a determining factor, since reduced thickness was associated with steep decreases in mechanical properties—a finding consistent with the present experimental data. Notably, Abdulmajeed et al.^9^ subjected their specimens to dynamic loading simulating chewing cycles—an approach that adds clinical validity by replicating realistic occlusal forces. Collectively, these results reinforce the need to consider not only chemical composition and manufacturing technology but also geometric parameters when designing zirconia restorations, thus optimizing their durability in functional environments.

 Differences between brands highlight the importance of selecting scientifically validated multilayer zirconias. For instance, Yucera showed significantly lower strength than Upcera and BeautiZir, possibly related to its microstructure or milling strategy. Nevertheless, further studies controlling variables such as layer position in the disk are needed, since milling closer to the “dentin” layer could optimize strength, and omission of thermocycling limits long-term extrapolation.

 The results of this study become especially relevant when contrasted with the biomechanical demands of the oral environment. Ting Sun et al.^15^ emphasize that monolithic zirconia crowns, due to their mechanical stability and high fracture load, are ideal for patients with parafunctional habits, where occlusal forces can exceed 1000 N. In the present study, even the brand with the lowest mean strength (Yucera: 1173 N) clearly surpasses the range of masticatory forces reported by Gélvez Vera et al.^5^ (133–727 N), suggesting that all three evaluated brands possess adequate load capacity to withstand functional demands in patients without severe parafunctions. However, in cases of bruxism or prolonged eccentric forces, brands such as BeautiZir (1807 N) or Upcera (1682 N) would offer a greater safety margin, consistent with Sun et al.’s^15^ recommendation to prioritize monolithic zirconia in high-risk contexts. These findings reinforce the clinical utility of multilayer zirconias, which combine layer-adapted strength (Li et al.,^17^ 2021) with gradient aesthetics without compromising restoration integrity.

 It is important to emphasize that the fracture load of multilayer zirconias does not depend solely on thickness or design but on a synergy of thermodynamic and microstructural factors. In addition to the yttria concentration—which increases the proportion of the translucent cubic phase but reduces toughness by suppressing the useful martensitic transformation—critical variables such as sintering protocols, cooling rates or rapid coolings that generate residual stress, and the distribution of crystalline phases (% tetragonal vs. cubic phase) determine the final mechanical performance. This multifactorial causality explains why Yucera®, with 8 layers of stratification and higher superficial translucency, exhibited lower strength than BeautyZir, which features 5 layers and lower translucency. Fundamentally, zirconias with a higher tetragonal phase preserve their stress-induced transformation mechanism: upon crack initiation, the tetragonal→monoclinic transition creates local volumetric expansion (~5%) that compresses the crack and dissipates energy, thereby increasing fracture toughness.^16^ When this mechanism is lost due to excess yttria, resulting in a dominant cubic phase—stable but brittle—the material relies solely on its intrinsic strength, resulting in premature failures under high occlusal loads. These findings underscore that optimizing multilayer zirconia requires balancing aesthetics through control of layer number and yttria percentage with thermomechanical integrity via careful sintering, gradual cooling, and preservation of a functional tetragonal phase.

## Conclusion

 This in vitro study demonstrated that monolithic multilayer zirconia crowns from the three evaluated brands (Yucera, Upcera, and BeautyZir) exhibited fracture loads sufficient to withstand the physiological occlusal forces reported in the literature (133–727 N), with mean values ranging from 1173 N(± 183) to 1807 N (± 463). However, statistically significant differences were observed between the brands, with Yucera showing the lowest strength (1173 N), which was significantly lower than Upcera (1682 N; *P* = 0.010) and BeautyZir (1807 N; *P* = 0.001). Moreover, no differences were found between Upcera and BeautyZir (*P* = 0.719), suggesting that both offer comparable mechanical performance under the conditions evaluated.

 The recorded standard deviations indicated greater variability in BeautyZir (± 463 N), possibly due to inconsistencies in the layer microstructure, variations during milling/sintering, or the milling position within the multilayer disc. In contrast, Yucera showed the lowest dispersion (± 183 N), indicating homogeneity that, paradoxically, did not result in greater strength.

 From a clinical perspective, these findings support the use of all three brands in patients with normal masticatory forces. However, in high-risk cases, Upcera and BeautyZir would be preferable options due to their greater safety margin.

## Recommendations

 Based on the results, the following recommendations are proposed:

- Although the use of crowns (instead of simplified specimens) aimed to replicate realistic clinical conditions, future studies should follow standardized protocols (e.g., ISO standards for dental ceramics) in specimen design and mechanical testing. This would facilitate comparison between studies without sacrificing clinical relevance.
- Incorporate thermocycling and cyclic loading (simulated mastication with equipment such as a “chewing simulator”) to evaluate fatigue resistance. This would replicate the progressive wear caused by humidity and repetitive forces and identify whether differences between brands persist after years of clinical use.
- The in vitro results should be validated with five-year in vivo follow-ups, monitoring fractures or wear in patients with crowns from the studied brands.
- The impact of adhesive cements on final compressive strength should be evaluated.
- Finite element analysis should be implemented to map stress and load distribution in zirconia crowns under simulated occlusal loading.

## Competing Interests

 The authors declare no conflicts of interest regarding the publication of this paper. No commercial or financial relationships with the manufacturers of the evaluated zirconia crowns influenced the design, implementation, analysis, or reporting of this study. All authors have reviewed and approved the final version of the manuscript.

## Ethical Approval

 The committee determined that “this study does not require ethical approval, as it does not involve human participants, biological specimens, or identifiable data”. The research was conducted entirely with zirconia test crowns, focusing on mechanical property evaluation. Therefore, it poses no ethical risks to human or animal subjects.

## References

[1] Arellano Moncayo AM, Peñate L, Arregui M, Giner-Tarrida L, Cedeño R (2023). State of the art of different zirconia materials and their indications according to evidence-based clinical performance: a narrative review. Dent J (Basel).

[2] Nevarez-Rascon A, González-Lopez S, Acosta-Torres LS, Nevarez-Rascon MM, Orrantia-Borunda E (2016). Synthesis, biocompatibility and mechanical properties of ZrO2-Al2O3 ceramics composites. Dent Mater J.

[3] Kang CM, Peng TY, Wu YA, Hsieh CF, Chi MC, Wu HY (2024). Comparison of optical properties and fracture loads of multilayer monolithic zirconia crowns with different yttria levels. J Funct Biomater.

[4] Solá-Ruíz MF, Rico-Coderch A, Montiel-Company JM, Fons-Badal C, Verdejo-Solá B, Agustín-Panadero R (2022). Influence of the chemical composition of monolithic zirconia on its optical and mechanical properties Systematic review and meta-regression. J Prosthodont Res.

[5] Gélvez Vera MA, Velosa-Porras J, Perez-Gutierrez B (2016). Effect of occlusal forces over periodontium analyzed through finite elements. Univ Odontol.

[6] Bustamante Fernández D. Efectos de Los Protocolos de Sinterización Y Simulación de Ajuste Oclusal en Las Propiedades Mecánicas y Confiabilidad de la Zirconia Multicapa Con Gradiente de Resistencia. San José: Universidad de Costa Rica; 2022. Available from: https://hdl.handle.net/10669/85816. Accessed August 17, 2024.

[7] Vallejo Panchez KG, Carrillo Vaca DG, Trujillo Jaramillo MC, Alban Hurtado CA, Salazar Martínez XG (2022). Zirconio monolítico su aplicación en estética dental. Tesla Rev Cient.

[8] Zhang Y, Chai H, Lee JJ, Lawn BR (2012). Chipping resistance of graded zirconia ceramics for dental crowns. J Dent Res.

[9] Abdulmajeed A, Sulaiman T, Abdulmajeed A, Bencharit S, Närhi T (2020). Fracture load of different zirconia types: a mastication simulation study. J Prosthodont.

[10] Lameira DP, Buarque e Silva WA, Andrade e Silva F, De Souza GM (2015). Fracture strength of aged monolithic and bilayer zirconia-based crowns. Biomed Res Int.

[11] Ministerio de Salud y Protección Social de Colombia. ENSAB IV. 2014. Available from: https://www.minsalud.gov.co/sites/rid/Lists/BibliotecaDigital/RIDE/VS/PP/ENSAB-IV-Situacion-Bucal-Actual.pdf. Accessed May 15, 2025.

[12] Duván Gutiérrez N, Galindo JAS, Yarpaz DC, Urbano V, Jiménez JF, León J (2025). [Oral health conditions and nutritional, functional and cognitive status - an approach to the institutionalized older adult]. Rev Esp Geriatr Gerontol.

[13] Martínez-Rus F, Pradíes Ramiro G, Suárez-García MJ, Rivera Gómez B (2007). Dental ceramics: classification and selection criteria. RCOE.

[14] Skjold A, Schriwer C, Øilo M (2019). Effect of margin design on fracture load of zirconia crowns. Eur J Oral Sci.

[15] Sun T, Zhou S, Lai R, Liu R, Ma S, Zhou Z (2014). Load-bearing capacity and the recommended thickness of dental monolithic zirconia single crowns. J Mech Behav Biomed Mater.

[16] Li QL, Jiang YY, Wei YR, Swain MV, Yao MF, Li DS (2022). The influence of yttria content on the microstructure, phase stability and mechanical properties of dental zirconia. Ceram Int.

[17] Li X, Wang Q, Qiu X, Zhao B (2021). Effect of different CAD/CAM cutting depths on the post-fatigue load-bearing capacity of novel multilayer zirconia restorations. J Dent.

[18] Honda J, Komine F, Kusaba K, Kitani J, Matsushima K, Matsumura H (2020). Fracture loads of screw-retained implant-supported zirconia prostheses after thermal and mechanical stress. J Prosthodont Res.

[19] Mellado Alfaro B, Anchelia Ramirez S, Quea Cahuana E (2015). Compressive resistance of lithium disilicate porcelain laminate veneers cemented with dual resin cement and self-adhesive dual resin cement in upper premolars. Int J Odontostomat.

[20] Nakamura T, Nakano Y, Usami H, Okamura S, Wakabayashi K, Yatani H (2020). In vitro investigation of fracture load and aging resistance of high-speed sintered monolithic tooth-borne zirconia crowns. J Prosthodont Res.

[21] Nakamura K, Harada A, Inagaki R, Kanno T, Niwano Y, Milleding P (2015). Fracture resistance of monolithic zirconia molar crowns with reduced thickness. Acta Odontol Scand.

